# Variable shift work in family medicine residents is associated with a non-dipper pattern and reduced phase angle: possible adaptive blood pressure mechanisms and body composition imbalance

**DOI:** 10.3389/fcvm.2026.1568573

**Published:** 2026-02-10

**Authors:** Elías Cardoso-Peña, José de Jesús Garduño-García, Alejandra Donají Benítez-Arciniega, Martha Beatriz Bustamante-Hernández, Rigoberto Oros-Pantoja, Alexandra E. Soto-Piña

**Affiliations:** 1 Unidad de Medicina Familiar 220, Instituto Mexicano del Seguro Social, Toluca, Mexico; 2 Facultad de Medicina, Universidad Autónoma del Estado de Mexico, Toluca, Mexico

**Keywords:** ABPM, blood pressure, non-dipper, phase angle, shift work, work stress

## Abstract

**Background:**

Shift work is associated with alterations in blood pressure (BP), metabolic changes, and unhealthy lifestyle patterns. However, the relationship between BP and variability in work hours remains poorly understood. Moreover, ambulatory blood pressure monitoring (ABPM) is a reliable approach to assess BP variability.

**Objective:**

The aim of this study was to evaluate whether shift work in family medicine residents (FMRs) modifies BP circadian patterns and whether these changes are related to body composition parameters.

**Methods:**

Thirty-eight FMRs were studied during 1 continuous 24-h day (D1) followed by 3 days of 6-h shifts (D2, D3, and D4). ABPM and body composition were assessed on D1 (postguard) and D4 (preguard). Systolic blood pressure (SBP) and diastolic blood pressure (DBP) were analyzed using nightly recordings from 10:00 p.m. to 3:00 a.m. Body composition was evaluated by electrical bioimpedance.

**Results:**

Of the 38 participants, 72.3% were women (30.2 ± 3.1 years old). Skeletal muscle mass (SMM) (24.0 ± 4.4 vs. 34.3 ± 6.2), *p* < 0.001, and total protein mass (TPM) (9.1 ± 2.0 vs. 11.8 ± 2), *p* < 0.001, were lower in women than in men. SBP showed significant positive correlations with SMM, fat mass (FM), fat mass at constant hydration, total body water, and TPM, while DBP was positively correlated only with TPM. On D1, the frequency of the non-dipper pattern was higher than the dipper *p* = 0.037. Furthermore, the phase angle (PhA) was significantly lower in the non-dipper group (6.8° ± 0.7°; *p* = 0.04) than the dipper group (7.4° ± 0.9°). DBP between 10 p.m. and 3 a.m. was lower (*p* = 0.010) on D4 (64.9 ± 6.3 mmHg) than on D1 (68.8 ± 5.2 mmHg).

**Conclusions:**

The non-dipper pattern was the most frequent in FMRs, and the reduction in PhA indicates that this type of nocturnal BP alteration could be related to CVD risk.

## 1 Introduction

Work stress has been associated with blood pressure (BP) alterations that could result in hypertension (1, 2) and other cardiovascular diseases (CVDs), which have been the leading cause of death worldwide since 2016 (3). Long working hours are linked to wakefulness, alertness, BP modifications, sleep deprivation, and metabolic disorders; in fact, job schedules with shift rotation have significant side effects (4–6). Other factors—including hereditary and immunological antecedents, poor diet, sedentary lifestyle, mental stress, extreme exhaustion (burnout), obesity, and gut microbiota imbalances—can affect BP regulation (6–9). Family medicine residents (FMRs) are especially vulnerable to cardiometabolic risk due to the demands of shift work and labor challenges required for their hospital activities, averaging between 88 and 98 h per week (10). In addition, sociodemographic variables such as marital status, family guardianship, specialty discipline, and degree of advancement can lead to burnout (11). However, there are not enough strategies to improve mental and physical health of medical residents.

Shift work can disrupt endocrinal circadian rhythms, affecting production of cortisol, insulin, leptin, and melatonin (12), with consequences for the cardiovascular system. For example, working 55 h or more per week increases the risk of cerebrovascular disease by 35% and ischemic cardiomyopathy-related mortality by 17% compared with a 35–40-h working week (13).

Ambulatory blood pressure monitoring (ABPM) is a reliable method for the diagnosis, follow-up, and treatment of high BP. It is also considered the most effective method for monitoring nighttime BP (14, 15) and is more accurate in predicting morbidity–mortality correlations than routine BP measurements (16). ABPM allows the classification of BP according to different nocturnal patterns (17). The normal or dipper pattern is defined by a decrease of more than 10% in nocturnal systolic blood pressure (SBP) at night compared with daytime or active periods (18–20). This decrease in BP is related to non-REM sleep, which lasts approximately 80% of the total sleep time (18, 21). The abnormal patterns include extreme dipper, non-dipper, and riser or reverse dipper, which are patterns associated with higher CVD risk (22). The extreme dipper pattern presents a decrease of ≥20%. The association of this pattern with CVD risk or organ damage remains unclear (23), but it may be relevant in older people (24). The non-dipper pattern is one where SBP decreases by 0% to 10% at night and it could be a risk factor for hypertension and non-fatal cardiovascular events (25). The riser or reverse dipper is one where BP increases at night instead of decreasing; this pattern has been reported in patients with hypertension, diabetes mellitus type 2, sleep apnea, stroke, and chronic kidney disease (23, 26).

Studies on ABPM in FMRs are scarce, although there is strong evidence on the association between the non-dipper pattern and CVD risk (14, 27). In particular, the non-dipper pattern is associated with mechanisms that disrupt the normal BP pattern, including perturbations in sleep patterns, autonomic dysfunction, water and sodium retention, hemodynamic alterations, and gut dysbiosis (17). Furthermore, the effects of medical work schedules on health have not shown a consensus. The lifestyle of medical professionals can promote the appearance of cardiometabolic alterations such as obesity, metabolic syndrome, diabetes, or other chronic diseases that are linked to low-grade chronic inflammation (28, 29). The effect of this systemic inflammatory state will affect each organ and lead to different morbidities (29).

Electrical bioimpedance provides body composition parameters and allows evaluation of the phase angle (PhA), which is the “reactance/resistance ratio” considered a novel biomarker of cell health. The reduction of PhA values is closely related to CVD risk (30) and inflammation (31). However, the relationship of PhA to CVD risk in medical residents—particularly in the context of high BP associated with variable work hours—remains unclear.

Therefore, the main aim of this study was to evaluate whether shift work in FMRs modifies patterns measured by ABPM and secondarily to assess body composition by electrical bioimpedance. These results will contribute to improving cardiovascular health and the work shift quality of medical residents.

## 2 Material and methods

An observational and prospective study was carried out among FMRs at the *Unidad de Medicina Familiar 220 del Instituto Mexicano del Seguro Social* (IMSS) (Family Medicine Unit 220, Social Security Mexican Institute). The sample included participants (24–38 years old) in their first to third academic year of residency, who completed all the questionnaires and measurements. Participants with previous diagnosis of hypertension, sleep and eating disorders, or under any pharmacological treatment for chronic disease were excluded. No sample size calculation was performed because all FMRs were included. Initially, 47 FMRs were enrolled in the study—34 women and 13 men—but only 38 completed it. Participant withdrawal from the study was mainly because some relocated to other medical units for their social service fulfillment, while others chose not to continue for personal reasons.

For the purpose of this analysis, the study included FMRs who followed a shift work scheme consisting of a continuous 24-h period in the hospital, during which they provided medical care in various services during the day and attended the emergency department at night. This was followed by 3 days of a non-continuous 6-h shift dedicated solely to medical care. The final sample comprised 38 FMRs, whose BP and body composition were measured. The study was approved by the 1503 Local Health Care Research Committee of IMSS; all FMRs provided informed consent to participate in the study.

### 2.1 Ambulatory blood pressure monitoring

An ambulatory blood pressure monitor device, model ABPM50 (Contec™), was used for 24-h BP monitoring for each participant. The equipment was calibrated before each measurement. On the first (D1) 24-h workday, starting at 8 a.m. and ending at 8 a.m. the following day, the first BP measurements were taken every 20 min during daytime (from 7 a.m. to 10 p.m.) and every 30 min during nighttime (from 10 p.m. to 7 a.m.), obtaining a total of 63 measurements in 24 h. D2 and D3 consisted of 6-h shifts starting at 08:00 and ending at 14:00, during which no measurements were performed. A second set of measurements was performed during the fourth 6-h shift day, from 8 a.m. to 2 p.m. (D4), and 18 h after that from 2 p.m. to 8 a.m. (recording of 24 h). Moreover, 30-min intervals of diastolic blood pressure (DBP) and SBP were recorded at night between 10 p.m. and 3 a.m. (11 measurements). An average of DBP and SBP values per every 30 min was computed for D1 and D4. Night SBP and DBP analyses were performed using recordings from 10:00 p.m. to 3:00 a.m.

### 2.2 Body composition evaluation

Body composition was assessed using electrical bioimpedance with the Biody Xpert^ZM^ II, (AMINOGram®, La Ciotat, France), a class IIa device under Central and Eastern Europe (CEE) law. First, height was measured by trained personnel using a stadimeter (SECA® 213, Seca Corporation) with a 20–205 cm range and body weight was measured with an Omron® scale model HBF-510LA (Omron®). Second, participant data—including the date of birth, name, sex, height, body weight, and physical activity—were entered into the Biody® application software; this information was required to automatically compute body composition parameters within the manufacturer’s application. Once each patient’s information was saved into the Biody application, the measurement stage was initiated by each participant holding the device, covering all four electrodes with the right hand and the top of the ankle, as specified by the manufacturer. Then, the measurement was performed, and the data were exported from the Biody® application. Computing of body composition parameters was based on a four-compartment model with multifrequency and multialgorithm analysis (32). In this case, the Biody Xpert^ZM^ II device employed five specific frequencies: 5 KHz for extracellular water (ECW), 20 KHz for extracellular fluid (ECF), 50 KHz for total body water (TBW), 100 KHz, a high frequency, able to penetrate the cell membranes and flow through both the intracellular fluid (ICF) and the ECF, and 250 KHz for body composition parameters. The data gathered at 250 KHz were used in conjunction with empirical equations and models (like the Cole model) to estimate various body composition parameters, including skeletal muscle mass (SMM), appendicular skeletal muscle Mass (ASMM), fat mass (FM), fat mass at constant hydration (FMH), total protein mass (TPM), phase angle (PhA), and body mass index (BMI). Moreover, at this kind of high frequency, the electrical current can pass through cell membranes and flow through both the intracellular water (ICW) and ECW. SMM was calculated by using the Skeletal Muscle Index (ASMM divided height squared) and the FFM ratio (fat mass/fat-free mass).

### 2.3 Glucose and glycated hemoglobin measurements

Glucose and hemoglobin (HbA1c) were measured after fasting for at least 8 h, before ABPM was applied at 8 a.m. on D1.

### 2.4 Statistical analysis

Quantitative data were expressed as mean ± standard deviation and qualitative variables were presented as absolute frequencies and percentages. The normality of data was assessed using the Kolmogorov–Simonov test. Student’s *t*-test was used to compare the mean differences in body composition variables and BP.

In addition, chi-square analysis was applied to identify differences between dipper and non-dipper percentages across D1 and D4 measurements of BP. Differences in nighttime SBP and DBP patterns (10 p.m.–3 a.m.) were assessed using Student’s *t*-test.

For all statistical tests, the level of significance considered was *p* < 0.050. Statistical analyses were performed using Statistical Package for Social Sciences (SPSS®), version 23 (SPSS Inc., Chicago, IL, USA).

## 3 Results

Table 1 summarizes body composition variables, BP, blood glucose, and HbA1c by sex. Of the 38 participants, 72.3% (*n* = 34) were women. The mean age was similar in both groups: 30.0 ± 3.4 years for the women and 30.6 ± 2.0 years for the men. There were no differences in BMI (26.4 ± 5.1 kg/m^2^ in women and 27.3 ± 5.7 kg/m^2^ in men), with both groups classified as overweight *p* = 0.620. In terms of body composition, SMM (34.3 ± 6.2 vs. 24.0 ± 4.4), *p* < 0.001, TPM (11.8 ± 2.0 vs. 9.1 ± 2.0), *p* < 0.001, and PhA (7.5 ± 0.8 vs. 6.7 ± 0.7), *p* = 0.008, were significantly higher in men. Glucose and HbA1c were within normal ranges. ABPM showed higher SBP, DBP, and MAP in men than in women during the 24-h measurements; however, these differences were not statistically significant.

**Table 1:** Body composition, blood metabolites, and BP of FMRs.

Variable	*N*	%	Mean	SD	*P*
Age (years)					
Men	13	27.6	30.6	2.0	0.560
Women	34	72.3	30.0	3.5	
BMI (kg/m^2^)					
Men	11	24.4	27.3	5.7	0.620
Women	34	75.6	26.4	5.1	
SMM (kg)					
Men	11	26.8	34.3	6.2	0.001*
Women	30	73.2	24.0	4.4	
FMH (%)					
Men	11	26.8	21.9	11.4	0.700
Women	30	73.2	23.3	9.7	
FM (kg)					
Men	11	26.8	19.3	12.3	0.147
Women	30	73.2	23.3	9.7	
TBW (L)					
Men	11	26.8	45.6	7.9	0.160
Women	30	73.2	33.0	4.8	
TPM (kg)					
Men	11	26.8	11.8	2.0	0.001*
Women	30	73.2	9.1	2.0	
PhA (°)					
Men	11	26.8	7.5	0.8	0.008*
Women	30	73.2	6.7	0.7	
Glucose (mg/dL)					
Men	10	29.4	95.1	18.1	0.530
Women	24	70.6	91.8	11.3	
HbA1c (%)					
Men	10	29.4	5.2	0.6	0.160
Women	24	70.6	4.9	0.4	
SBP (mmHg)					
Men	13	27.7	117.5	6.0	0.070
Women	34	72.3	112.9	8.6	
DBP (mmHg)					
Men	13	27.7	73.2	6.6	0.200
Women	34	72.3	70.3	6.9	
MBP (mmHg)					
Men	13	27.7	88.1	6.2	0.120
Women	34	72.3	84.4	7.3	
Heart rate (BPM)					
Men	13	27.7	79.7	8.4	0.670
Women	34	72.3	78.6	7.7	

Values are shown as frequencies, percentages, and mean ± SD. BMI, body mass index; SMM, skeletal muscle mass; FM, fat mass; FMH, fat mass at constant hydration; TBW, total body water; TPM, total protein mass; PhA, phase angle; HbA1c, glycosylated hemoglobin; SBP, systolic blood pressure; DBP, diastolic blood pressure; MAP, mean arterial pressure (averages of 24-h ABPM recordings).

* Statistical significance level was considered as *p* < 0.05 for Student’s *t*-test.

A positive and significant correlation was observed between SBP and SMM (*r* = 0.4, *p* = 0.014), FM (*r* = 0.3, *p* = 0.016), FMH (*r* = 0.3, *p* = 0.032), TBW (*r* = 0.3, *p* = 0.019), and TPM (*r* = 0.5, *p* = 0.001). DBP showed a positive correlation only with TPM (*r* = 0.4, *p* = 0.012) (Table 2).

**Table 2:** Correlation between BP and body composition.

Variable	SMM (kg)		FM (kg)		FMH (%)		TBW (L)		TPM (kg)		PhA (°)	
		*p*		*p*		*p*		*p*		*p*		*p*
SBP	0.4	0.014*	0.3	0.016*	0.3	0.032*	0.3	0.019*	0.5	0.001*	0.1	0.817
DBP	0.3	0.013*	0.3	0.065	0.2	0.160	0.2	0.141	0.4	0.012*	−0.1	0.836

SMM, skeletal muscle mass; FM, fat mass; FMH, fat mass at constant hydration; TBW, total body water; TPM, total protein mass; PhA, phase angle; SBP, systolic blood pressure; DBP, diastolic blood pressure. Values represent Pearson correlation coefficients.

* A correlation is considered significant at *p* < 0.05.

No significant differences were observed between dipper and non-dipper groups in SMM, FM, FMH, TBW, or TPM (*p* > 0.050). Nonetheless, PhA was significantly higher in the dipper group compared with the non-dipper group (7.4 ± 0.9 vs. 6.8 ± 0.7), *p* = 0.040 (Table 3).

**Table 3:** Body composition and phase angle according to dipper pattern in FMRs.

Variable	Dipper		No dipper		*p*
	Mean	SD	Mean	SD	
SMM (kg)	26.3	5.3	26.9	7.1	0.79
FMH (%)	20.2	10.1	23.8	10.1	0.33
FM (kg)	16.9	6.5	24.1	11.0	0.16
TBW (L)	35.6	5.9	36.6	8.6	0.74
TPM (kg)	9.6	2.1	9.9	2.3	0.67
PhA (°)	7.4	0.9	6.8	0.7	0.04*

SMM, skeletal muscle mass; FM, fat mass; FMH, fat mass at constant hydration; TBW, total body water; TPM, total protein mass; PhA, phase angle. Values are shown as mean ± SD.

* Statistical significance level was considered as *p* < 0.05 for Student’s *t*-test.

When analyzing the BP patterns (Table 4), the most prevalent in D1 was the non-dipper pattern (a nighttime SBP reduction of 0–10 mmHg) (57.4%). This was followed by the inverse dipper pattern (a nighttime SBP increase) (19.1%) and the dipper pattern (a nighttime reduction in SBP of 10–20 mmHg) (17.0%). However, in D4, the pattern percentages were different from D1; the dipper pattern became the most frequent (39.5%), followed by the non-dipper (36.5%) and inverse dipper (21.1%).

**Table 4:** Blood pressure patterns according to D1 and D4.

BP pattern	D1		D4		*p*
	*N*	%	*N*	%	
Non-dipper	27	57.4	14	36.8	0.085
Dipper	8	17.0	15	39.5	
Extreme dipper	3	6.4	1	2.6	
Inverse dipper	9	19.1	8	21.1	

Values are expressed as frequencies and percentages of 24-h measurements, non-dipper blood pressure drop between 0% and 10%, dipper pattern drop between 10% and 20%, extreme dipper drop >20%, and inverse dipper increase at least 1 mmHg in BP. Statistical significance level was considered as *p* < 0.05 for chi-square analysis.

Moreover, FMRs were categorized into two distinct groups: dipper and non-dipper patterns. The non-dipper pattern was more frequent in D1 (*n* = 36) than the dipper one (*n* = 11). In D4, the non-dipper frequency decreased (*n* = 22), while the dipper frequency increased (*n* = 17). In addition, a statistically significant association was found between the non-dipper frequency and D1 (*p* = 0.037). In men, the non-dipper pattern in D1 was more frequent (*n* = 9) than the dipper (*n* = 4), but it was less frequent in D4 (2.5 *p* = 0.110). In women, the non-dipper pattern was more frequent in D1 (*n* = 27) than the dipper (*n* = 7); in D4, the non-dipper (*n* = 16) was lower than in D1 (*p* = 0.180).

**Figure 1:**
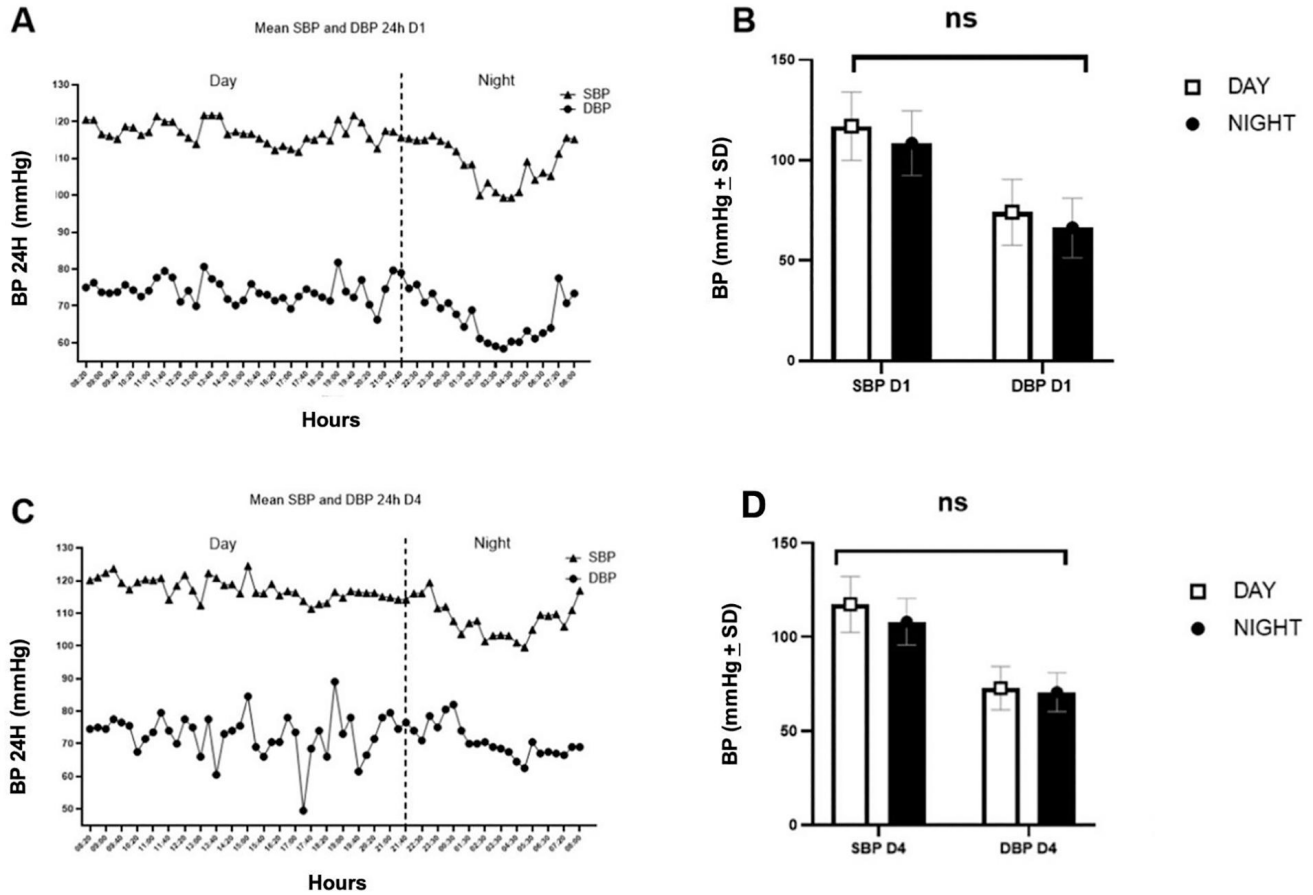
**(A,B)** Mean SBP and DBP of FMRs, 24 h D1. **(C,D)** Mean SBP and DBP of FMRs, 24 h D4. DBP, diastolic blood pressure; SBP, systolic blood pressure, *p < 0.05, Student’s *t*-test.

Figures 1A,B illustrate a decreasing trend in SBP and DBP during nighttime (after 10:00 p.m.) compared with daytime values; however, no statistically significant differences were observed (Figures 1C,D).

**Figure 2:**
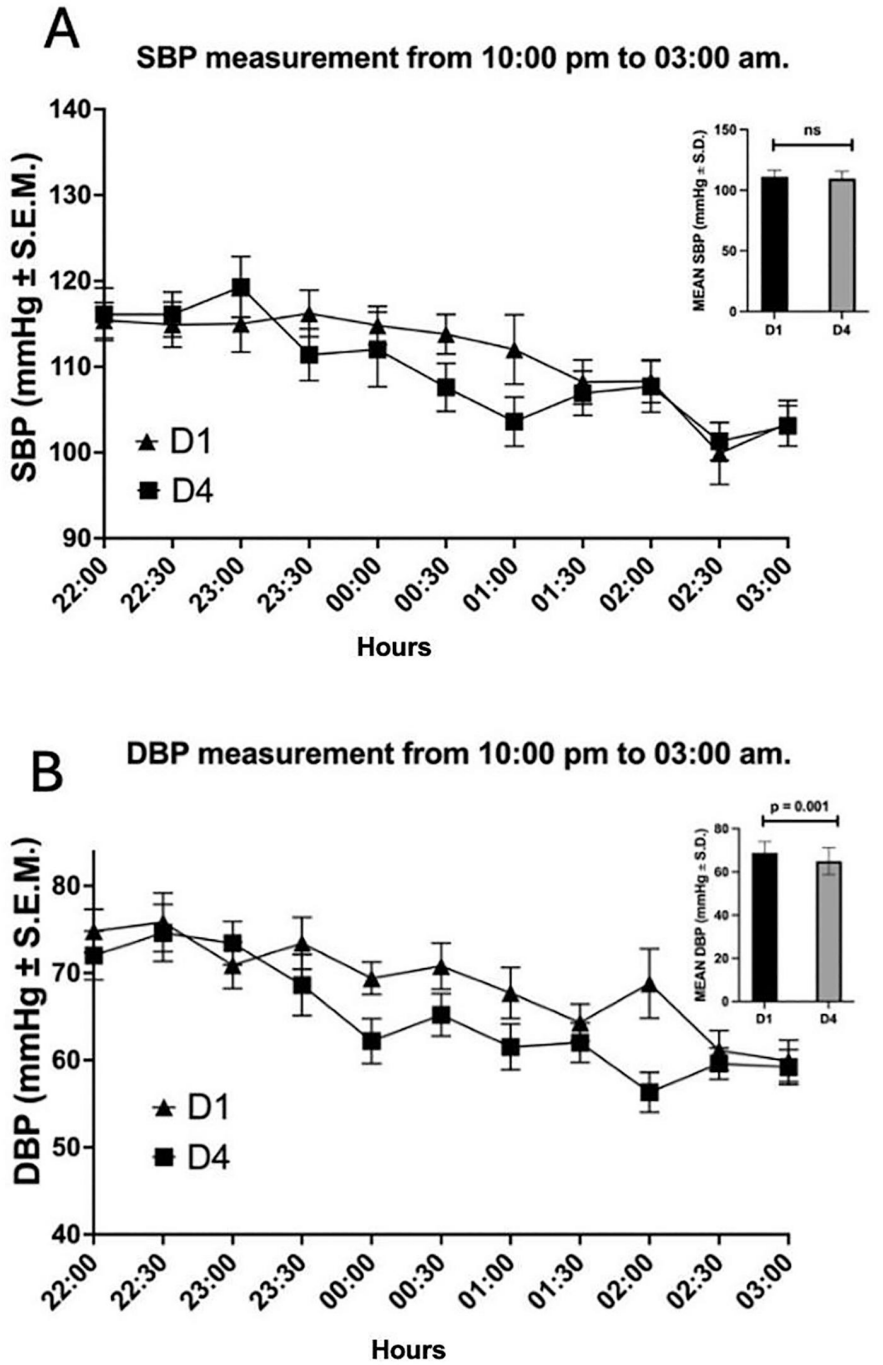
Mean SBP and DBP of FMRs, 10:00 p.m. to 3:00 a.m. DBP, diastolic blood pressure **(A)**; SBP, systolic blood pressure **(B)**. The difference between the means of D1 and D4 is represented. DBP (*p* = 0.01) and SBP (*p* = 0.20), Student’s *t*-test.

 DBP between 10 p.m. and 3 a.m. (Figure 2A) was significantly lower in D4 (64.9 ± 6.2 mmHg) than D1 (68.8 ± 5.2 mmHg, *p* = 0.010). However, there were no differences in SBP between D1 (111.1 ± 5.4 mmHg) and D4 (109.5 ± 5.9 mmHg, *p* = 0.200) (Figure 2B).

## 4 Discussion

This investigation revealed that the non-dipper pattern in FMRs was more frequent in D1 than in D4, suggesting that 24-h shift work may be associated with increases in BP and potentially with hypertension and CVD risk. Although the differences were more evident in the postguard stage, in most cases, the changes were not adaptive during the non-guard (D2–D3) and preguard (D4) periods. These findings align closely with previous reports showing that night shifts increase SBP and DBP and reduce the dipping pattern in internists (33). Furthermore, factors such as long working hours (34), age (>50 years old) (35), and having 5 or more night shifts per month augment hypertension prevalence (36). In our study, the average age of the participants was 30 and demonstrated an increase in SBP and DBP of about 1 mmHg between D1 and D4. Moreover, the workload burden in this FMR group is considerable because their residency training usually involves 24-h hospital shifts every 4 days, possibly extending up to 8 years.

This type of workload can be related to CVD risk. For example, a 10-h work shift has been shown to increase the risk of ischemic heart disease and heart attack over a period of 10 years or more (37). The shift work of FMRs in our study can reach 36 continuous hours or more every 4 days. Therefore, our findings, along with other reports (38–42), suggest that the risk and prevalence of CVD, diabetes mellitus, kidney failure, retinopathy, and other chronic conditions could significantly increase in this population. Interestingly, this misalignment between the work/rest periods and the circadian rhythm is known as social jetlag and is considered a predictor of CVD risk (43).

Night shift workers are more prone to develop diabetes mellitus type 2 and metabolic syndrome risk (44, 45). Furthermore, night shift work is associated with increased blood glucose concentrations (46). For instance, in Korean night shift workers, fasting glucose was 101.0 ± 21.5 mg/dL (47), but in our FMR sample, it was lower (95.1 ± 18.1 mg/dL in men and 91.8 ± 11.3 in women). Although the levels of glucose and HbA1c were within normal ranges in our sample, fasting glucose was close to the upper limit.

Body composition analysis revealed that FMH in men was slightly above the normal level (21.9%). However, among women, FMH was lower (23.3%). Moreover, values of SMM were within the normal category for the Mexican population (48). However, SMM and TPM were significantly lower in women than in men, consistent with prior reports on the Mexican population (48). These differences are related to the physiological and hormonal features of each sex. Social jetlag can affect SMM by disrupting hormonal homeostasis, producing inflammation and disrupting muscle metabolism (49). Furthermore, the disruption of the biological clock that regulates muscle health involves protein intake, exercise, and hormone balance (50). Shift work has also been associated with sarcopenia and higher total energy intake (51). We did not measure food consumption or physical activity in this study, which represents a limitation in determining whether these factors could influence SMM in FMRs.

SBP was positively correlated with SMM, FM, FMH, TBW, and TPM, with the strongest association observed for TPM. This correlation could be explained, in part, by the action of irisin, a hormone produced by muscle, which has been linked to increased blood pressure (52). DBP correlated only with TPM, suggesting a specific role of protein mass in diastolic regulation (52). Interestingly, we found that residents with non-dipper BP presented a lower PhA than residents with dipper patterns. This is in agreement with other reports where PhA has been considered an indirect marker of CVD risk (53, 54). Therefore, PhA could be a sensitive tool for monitoring CVD risk and could be relevant for prevention strategies. Moreover, PhA was lower in women than in men, which coincides with the lower SMM and TPM in women.

Although dual-energy X-ray absorptiometry (DEXA) is considered the most accurate method for estimating body composition, multifrequency bioimpedance analysis offers high reliability despite being an indirect method. However, it has certain disadvantages and limitations that must be considered. For example, bioimpedance can be affected by the patient’s hydration status, physical activity, and ambient temperature (55). Furthermore, the body composition data obtained from the Biody Xpert^ZM^ II device uses multiple algorithms; therefore, most of these results (FM, TPM, mineral content, metabolic risks, cardiovascular risks, etc.) are derived from prediction equations that must be adjusted for specific study populations according to ethnicity and age. These factors can specifically affect the accuracy of PhA, underscoring the need for standardization protocols among manufacturers (56) and validation with biochemical markers (e.g., metabolic and inflammatory).

It is important to consider the role of other biomarkers in BP dysregulation due to night shifts. The expression of muscle and hepatic clock genes and the measurement of cortisol are necessary to understand the role of peripheral clock disruption (46, 57, 58). We did not evaluate food consumption, lifestyle habits, or sleep disorders in our participants, which limits conclusions related to the BP dipping patterns and characterization of social jetlag in FMRs. This study was restricted to FMRs in their first to third years of training. However, future studies should include other on-call rotation schedules, medical residencies, and follow-up of postgraduate medical resident students.

The short- and long-term effects of shift work during the medical training period (and after graduation) have not been fully evaluated. Although work stress varies by specialty and improvements are implemented regularly, challenges remain constant (59). According to the American Medical Association (AMA), Family Medicine is among the medical specialties with the highest rates of burnout due to its primary care role, long working hours, administrative demands, and mental fatigue. For example, emergency department residents show higher SBP and DBP during sleep in 24-h shifts compared with a typical workday (60). In Korean residents across multiple medical specialties, the first 2 years of training showed the greatest decompensation on BMI, BP, liver function test results, and total cholesterol (61). This was attributed to greater work pressure as well as longer working hours (≥80 h). Reports on resident graduates are scarce, highlighting the need for longitudinal and follow-up studies to draw specific conclusions and establish workplace health programs.

At the hospital where the study was conducted, policies exist regarding the health status of medical residents. However, dissemination of research on the occupational risks of shift work—in addition to work-related stress itself—is crucial. Our results show that FMRs exhibit a prevalent non-dipper nighttime BP pattern, which may be related to hypertension and CVD risk. Institutional strategies to improve shift work can include adding an extra day off between shifts and ensuring appropriate schedules for eating, hydration, and rest, which are associated with reduced cardiovascular alterations and CVD risk (62, 63). It is also essential to promote changes in lifestyle, such as healthier diets, physical activity, and improved sleep habits among medical residents.

## Data Availability

The raw data supporting the conclusions of this article will be made available by the authors, without undue reservation.
